# Dibromidobis(triphenyl­arsine)palladium(II)

**DOI:** 10.1107/S1600536809001986

**Published:** 2009-01-23

**Authors:** Leo Kirsten, Gideon Steyl

**Affiliations:** a Department of Chemistry, University of the Free State, PO Box 339, Bloemfontein 9300, South Africa

## Abstract

In the title compound, [PdBr_2_(C_18_H_15_As)_2_], the Pd^II^ ion resides on a centre of symmetry and is coordinated by two As atoms [Pd—As = 2.4184 (3) Å] and two Br anions [Pd—Br = 2.4196 (3) Å] in a slightly distorted square-planar geometry [As—Pd—Br = 90.12 (1)°]. The crystal packing exhibits weak inter­molecular C—H⋯Br inter­actions.

## Related literature

For similar palladium structures containing triphenyl­phosphine and bromido moieties, see: Crawforth *et al.* (2005 ▶); Stark & Whitmire (1997 ▶); Rodriguez *et al.* (2007 ▶). For the crystal structures of related bromido arsine complexes, see: Singh *et al.* (1999 ▶); Phadnis *et al.* (2003*a* ▶,*b* ▶).
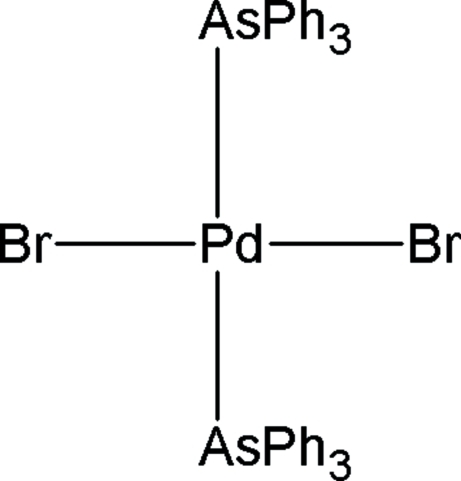


## Experimental

### 

#### Crystal data

[PdBr_2_(C_18_H_15_As)_2_]
*M* *_r_* = 878.66
Monoclinic, 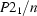

*a* = 9.3754 (11) Å
*b* = 19.545 (3) Å
*c* = 9.8151 (13) Å
β = 112.798 (3)°
*V* = 1658.1 (4) Å^3^
*Z* = 2
Mo *K*α radiation
μ = 4.97 mm^−1^
*T* = 100 (2) K
0.32 × 0.23 × 0.18 mm

#### Data collection

Bruker APEXII CCD diffractometer
Absorption correction: multi-scan (*SADABS*; Bruker, 1998 ▶) *T*_min_ = 0.264, *T*_max_ = 0.408
18511 measured reflections
3619 independent reflections
3245 reflections with *I* > 2σ(*I*)
*R*_int_ = 0.028

#### Refinement

*R*[*F* ^2^ > 2σ(*F* ^2^)] = 0.019
*wR*(*F* ^2^) = 0.043
*S* = 1.04
3619 reflections
187 parameters
8 restraints
H-atom parameters constrained
Δρ_max_ = 0.50 e Å^−3^
Δρ_min_ = −0.34 e Å^−3^

### 

Data collection: *APEX2* (Bruker, 2005 ▶); cell refinement: *SAINT-Plus* (Bruker, 2004 ▶); data reduction: *SAINT-Plus* and *XPREP* (Bruker 2004 ▶); program(s) used to solve structure: *SHELXS97* (Sheldrick, 2008 ▶); program(s) used to refine structure: *SHELXL97* (Sheldrick, 2008 ▶); molecular graphics: *DIAMOND* (Brandenburg & Putz, 2006 ▶); software used to prepare material for publication: *SHELXL97*.

## Supplementary Material

Crystal structure: contains datablocks I, global. DOI: 10.1107/S1600536809001986/cv2489sup1.cif

Structure factors: contains datablocks I. DOI: 10.1107/S1600536809001986/cv2489Isup2.hkl

Additional supplementary materials:  crystallographic information; 3D view; checkCIF report

## Figures and Tables

**Table 1:** Hydrogen-bond geometry (Å, °)

*D*—H⋯*A*	*D*—H	H⋯*A*	*D*⋯*A*	*D*—H⋯*A*
C13—H13⋯Br^i^	0.95	2.90	3.807 (3)	160
C25—H25⋯Br^ii^	0.95	2.98	3.914 (3)	168

Symmetry codes: (i) 
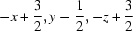
; (ii) 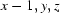.

**Fig. 1.**
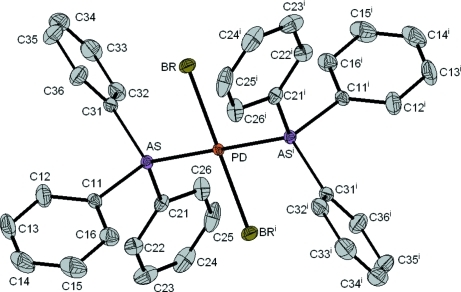
Molecular structure of (I) showing the atomic numbering and 50% probability displacement ellipsoids [symmetry code: (i) 1-x, 1-y, 1-z]. Hydrogen atoms have been omitted for clarity.

## supplementary crystallographic information

### Comment

Palladium complexes containing phosphine and bromo derivatives have been investigated in the past (Crawforth *et al.*, 2005; Stark *et al.*, 1997; Rodriguez *et al.*, 2007). The effect of phosphine substitution by arsine moieties in these complexes have received limited attention. Up to date the structures of only a few bromo arsine complexes have been characterized (Singh *et al.*, 1999; Phadnis *et al.*, 2003*a*; Phadnis *et al.*, 2003*b*).

The title compound, (I), crystallizes in the P2_1_/n space group with the Pd atom on a centre of symmetry (0.5, 0.5, 0.5). A staggered conformation of the two triphenyl arsine fragments is supported by the Br—Pd—As—Cn torsion angles of -98.07 (6)° (Cn=C11), 146.61 (7)° (Cn=C21) and 22.87 (7)° (Cn=C31), respectively. A weak intermolecular interaction is observed between the bromo moiety and the hydrogen atoms of the triphenylarsine ligand (Table 2).

### Experimental

The title compound was synthesized by the addition of AsPh3 (17 mg, 0.0059 mmol) to an acetone solution (15 cm^3^) of Pd(Br)_2_(COD) (10 mg, 0.027 mmol). Crystals suitable for diffraction were obtained by slow evaporation of the reaction mixture (yield 15 mg, 64%).

### Refinement

All H atoms were positioned geometrically and allowed to ride on their parent atoms, with C—H = 0.95 Å and *U*_iso_(H) = 1.2*U*_eq_(parent).

### Crystal data

**Table:**

[PdBr_2_(C_18_H_15_As)_2_]	*F*(000) = 856
*M**_r_* = 878.66	*D*_x_ = 1.760 Mg m^−^^3^
Monoclinic, *P*2_1_/*n*	Mo *K*α radiation, λ = 0.71073 Å
Hall symbol: -P 2yn	Cell parameters from 8105 reflections
*a* = 9.3754 (11) Å	θ = 2.5–28.3°
*b* = 19.545 (3) Å	µ = 4.97 mm^−^^1^
*c* = 9.8151 (13) Å	*T* = 100 K
β = 112.798 (3)°	Cuboid, orange
*V* = 1658.1 (4) Å^3^	0.32 × 0.23 × 0.18 mm
*Z* = 2	

### Data collection

**Table:**

Bruker X8 APEXII 4K KappaCCD diffractometer	3619 independent reflections
Radiation source: fine-focus sealed tube	3245 reflections with *I* > 2σ(*I*)
graphite	*R*_int_ = 0.028
Detector resolution: 512 pixels mm^-1^	θ_max_ = 27.0°, θ_min_ = 2.1°
φ and ω scans	*h* = −10→11
Absorption correction: multi-scan (*SADABS*; Bruker, 1998)	*k* = −23→24
*T*_min_ = 0.264, *T*_max_ = 0.408	*l* = −12→11
18511 measured reflections	

### Refinement

**Table:**

Refinement on *F*^2^	Primary atom site location: structure-invariant direct methods
Least-squares matrix: full	Secondary atom site location: difference Fourier map
*R*[*F*^2^ > 2σ(*F*^2^)] = 0.019	Hydrogen site location: inferred from neighbouring sites
*wR*(*F*^2^) = 0.043	H-atom parameters constrained
*S* = 1.04	*w* = 1/[σ^2^(*F*_o_^2^) + (0.0179*P*)^2^ + 0.8574*P*] where *P* = (*F*_o_^2^ + 2*F*_c_^2^)/3
3619 reflections	(Δ/σ)_max_ = 0.001
187 parameters	Δρ_max_ = 0.50 e Å^−^^3^
8 restraints	Δρ_min_ = −0.34 e Å^−^^3^

### Special details

**Table:**

Geometry. All e.s.d.'s (except the e.s.d. in the dihedral angle between two l.s. planes) are estimated using the full covariance matrix. The cell e.s.d.'s are taken into account individually in the estimation of e.s.d.'s in distances, angles and torsion angles; correlations between e.s.d.'s in cell parameters are only used when they are defined by crystal symmetry. An approximate (isotropic) treatment of cell e.s.d.'s is used for estimating e.s.d.'s involving l.s. planes.
Refinement. Refinement of *F*^2^ against ALL reflections. The weighted *R*-factor *wR* and goodness of fit *S* are based on *F*^2^, conventional *R*-factors *R* are based on *F*, with *F* set to zero for negative *F*^2^. The threshold expression of *F*^2^ > σ(*F*^2^) is used only for calculating *R*-factors(gt) *etc*. and is not relevant to the choice of reflections for refinement. *R*-factors based on *F*^2^ are statistically about twice as large as those based on *F*, and *R*- factors based on ALL data will be even larger.

### Fractional atomic coordinates and isotropic or equivalent isotropic displacement parameters (Å^2^)

**Table:**

	*x*	*y*	*z*	*U*_iso_*/*U*_eq_	
Pd	0.5000	0.5000	0.5000	0.01115 (5)	
As	0.47028 (2)	0.402209 (10)	0.63768 (2)	0.01207 (5)	
C11	0.5474 (2)	0.32083 (11)	0.5777 (2)	0.0159 (4)	
C12	0.6082 (3)	0.26633 (13)	0.6727 (3)	0.0326 (6)	
H12	0.6178	0.2690	0.7725	0.039*	
C13	0.6551 (3)	0.20769 (14)	0.6211 (3)	0.0433 (7)	
H13	0.6980	0.1705	0.6865	0.052*	
C14	0.6401 (3)	0.20317 (14)	0.4774 (3)	0.0415 (7)	
H14	0.6716	0.1628	0.4428	0.050*	
C15	0.5795 (3)	0.25703 (15)	0.3832 (3)	0.0409 (7)	
H15	0.5689	0.2539	0.2831	0.049*	
C16	0.5337 (2)	0.31601 (12)	0.4331 (2)	0.0251 (5)	
H16	0.4928	0.3533	0.3674	0.030*	
C21	0.2577 (2)	0.37831 (11)	0.6001 (2)	0.0165 (4)	
C22	0.2010 (2)	0.31345 (12)	0.5546 (2)	0.0210 (5)	
H22	0.2666	0.2792	0.5419	0.025*	
C23	0.0471 (3)	0.29836 (14)	0.5275 (2)	0.0300 (6)	
H23	0.0067	0.2542	0.4936	0.036*	
C24	−0.0461 (3)	0.34754 (15)	0.5498 (3)	0.0343 (6)	
H24	−0.1500	0.3367	0.5342	0.041*	
C25	0.0092 (3)	0.41231 (15)	0.5943 (3)	0.0359 (6)	
H25	−0.0564	0.4460	0.6092	0.043*	
C26	0.1618 (3)	0.42845 (13)	0.6177 (3)	0.0269 (5)	
H26	0.1998	0.4735	0.6454	0.032*	
C31	0.5682 (2)	0.40056 (11)	0.8519 (2)	0.0181 (4)	
C32	0.4806 (3)	0.40942 (11)	0.9370 (2)	0.0250 (5)	
H32	0.3719	0.4159	0.8900	0.030*	
C33	0.5505 (3)	0.40885 (13)	1.0895 (3)	0.0357 (6)	
H33	0.4899	0.4151	1.1469	0.043*	
C34	0.7067 (4)	0.39928 (14)	1.1579 (3)	0.0411 (7)	
H34	0.7540	0.3982	1.2628	0.049*	
C35	0.7966 (3)	0.39115 (14)	1.0751 (3)	0.0397 (7)	
H35	0.9052	0.3850	1.1233	0.048*	
C36	0.7278 (3)	0.39204 (13)	0.9216 (3)	0.0288 (5)	
H36	0.7891	0.3869	0.8646	0.035*	
Br	0.73567 (2)	0.531376 (11)	0.70471 (2)	0.01978 (6)	

### Atomic displacement parameters (Å^2^)

**Table:**

	*U*^11^	*U*^22^	*U*^33^	*U*^12^	*U*^13^	*U*^23^
Pd	0.01348 (10)	0.00966 (11)	0.01033 (10)	−0.00012 (8)	0.00462 (8)	0.00050 (8)
As	0.01394 (9)	0.01108 (11)	0.01179 (10)	0.00054 (7)	0.00563 (7)	0.00151 (8)
C11	0.0129 (9)	0.0133 (11)	0.0210 (10)	0.0004 (8)	0.0059 (8)	−0.0019 (8)
C12	0.0415 (13)	0.0221 (13)	0.0324 (13)	0.0100 (11)	0.0123 (11)	0.0061 (10)
C13	0.0366 (14)	0.0201 (14)	0.0622 (19)	0.0120 (11)	0.0070 (13)	0.0035 (13)
C14	0.0289 (13)	0.0295 (16)	0.0652 (19)	0.0042 (11)	0.0171 (13)	−0.0202 (14)
C15	0.0450 (15)	0.0405 (17)	0.0433 (16)	0.0013 (13)	0.0236 (13)	−0.0184 (13)
C16	0.0283 (11)	0.0247 (13)	0.0247 (12)	0.0017 (10)	0.0129 (9)	−0.0042 (10)
C21	0.0147 (9)	0.0209 (11)	0.0150 (10)	0.0005 (8)	0.0068 (7)	0.0053 (8)
C22	0.0227 (10)	0.0250 (13)	0.0178 (10)	−0.0031 (9)	0.0103 (8)	0.0015 (9)
C23	0.0256 (11)	0.0405 (16)	0.0227 (12)	−0.0133 (11)	0.0079 (9)	0.0030 (10)
C24	0.0190 (11)	0.0521 (18)	0.0313 (13)	−0.0023 (11)	0.0094 (10)	0.0178 (12)
C25	0.0258 (12)	0.0480 (18)	0.0398 (14)	0.0205 (12)	0.0192 (11)	0.0218 (13)
C26	0.0267 (11)	0.0240 (13)	0.0342 (13)	0.0056 (10)	0.0163 (10)	0.0084 (10)
C31	0.0271 (10)	0.0127 (11)	0.0124 (10)	−0.0017 (8)	0.0053 (8)	0.0016 (8)
C32	0.0399 (13)	0.0180 (12)	0.0194 (11)	−0.0010 (10)	0.0141 (10)	0.0016 (9)
C33	0.0635 (17)	0.0268 (14)	0.0209 (12)	−0.0001 (12)	0.0209 (12)	0.0006 (10)
C34	0.0724 (19)	0.0254 (14)	0.0150 (11)	−0.0048 (13)	0.0054 (12)	0.0019 (10)
C35	0.0384 (14)	0.0359 (16)	0.0276 (13)	−0.0009 (12)	−0.0061 (11)	0.0058 (11)
C36	0.0268 (11)	0.0312 (14)	0.0227 (12)	0.0019 (10)	0.0036 (9)	0.0056 (10)
Br	0.01837 (10)	0.02032 (12)	0.01603 (10)	−0.00379 (8)	0.00159 (8)	0.00096 (8)

### Geometric parameters (Å, °)

**Table:**

Pd—As^i^	2.4184 (3)	C22—C23	1.394 (3)
Pd—As	2.4184 (3)	C22—H22	0.9500
Pd—Br	2.4196 (3)	C23—C24	1.372 (4)
Pd—Br^i^	2.4196 (3)	C23—H23	0.9500
As—C11	1.931 (2)	C24—C25	1.374 (4)
As—C21	1.9383 (19)	C24—H24	0.9500
As—C31	1.942 (2)	C25—C26	1.395 (3)
C11—C16	1.378 (3)	C25—H25	0.9500
C11—C12	1.385 (3)	C26—H26	0.9500
C12—C13	1.391 (4)	C31—C32	1.391 (3)
C12—H12	0.9500	C31—C36	1.393 (3)
C13—C14	1.365 (4)	C32—C33	1.382 (3)
C13—H13	0.9500	C32—H32	0.9500
C14—C15	1.371 (4)	C33—C34	1.367 (4)
C14—H14	0.9500	C33—H33	0.9500
C15—C16	1.384 (3)	C34—C35	1.389 (4)
C15—H15	0.9500	C34—H34	0.9500
C16—H16	0.9500	C35—C36	1.390 (3)
C21—C22	1.380 (3)	C35—H35	0.9500
C21—C26	1.385 (3)	C36—H36	0.9500
			
As^i^—Pd—As	180.000 (6)	C21—C22—C23	119.8 (2)
As^i^—Pd—Br	89.876 (10)	C21—C22—H22	120.1
As—Pd—Br	90.124 (10)	C23—C22—H22	120.1
As^i^—Pd—Br^i^	90.124 (10)	C24—C23—C22	119.8 (2)
As—Pd—Br^i^	89.876 (10)	C24—C23—H23	120.1
Br—Pd—Br^i^	180.0	C22—C23—H23	120.1
C11—As—C21	102.85 (9)	C23—C24—C25	120.8 (2)
C11—As—C31	103.93 (9)	C23—C24—H24	119.6
C21—As—C31	102.82 (8)	C25—C24—H24	119.6
C11—As—Pd	110.05 (6)	C24—C25—C26	119.8 (2)
C21—As—Pd	114.63 (6)	C24—C25—H25	120.1
C31—As—Pd	120.65 (6)	C26—C25—H25	120.1
C16—C11—C12	119.4 (2)	C21—C26—C25	119.6 (2)
C16—C11—As	118.30 (16)	C21—C26—H26	120.2
C12—C11—As	122.23 (17)	C25—C26—H26	120.2
C11—C12—C13	119.6 (2)	C32—C31—C36	119.5 (2)
C11—C12—H12	120.2	C32—C31—As	120.45 (16)
C13—C12—H12	120.2	C36—C31—As	120.05 (16)
C14—C13—C12	120.6 (3)	C33—C32—C31	120.4 (2)
C14—C13—H13	119.7	C33—C32—H32	119.8
C12—C13—H13	119.7	C31—C32—H32	119.8
C13—C14—C15	119.8 (2)	C34—C33—C32	120.1 (2)
C13—C14—H14	120.1	C34—C33—H33	119.9
C15—C14—H14	120.1	C32—C33—H33	119.9
C14—C15—C16	120.3 (2)	C33—C34—C35	120.4 (2)
C14—C15—H15	119.8	C33—C34—H34	119.8
C16—C15—H15	119.8	C35—C34—H34	119.8
C11—C16—C15	120.2 (2)	C34—C35—C36	120.1 (2)
C11—C16—H16	119.9	C34—C35—H35	120.0
C15—C16—H16	119.9	C36—C35—H35	120.0
C22—C21—C26	120.19 (19)	C35—C36—C31	119.5 (2)
C22—C21—As	121.45 (16)	C35—C36—H36	120.2
C26—C21—As	118.35 (17)	C31—C36—H36	120.2
			
Br—Pd—As—C11	−98.07 (6)	C31—As—C21—C26	78.50 (18)
Br^i^—Pd—As—C11	81.93 (6)	Pd—As—C21—C26	−54.30 (17)
Br—Pd—As—C21	146.61 (7)	C26—C21—C22—C23	−0.5 (3)
Br^i^—Pd—As—C21	−33.39 (7)	As—C21—C22—C23	−179.43 (15)
Br—Pd—As—C31	22.87 (7)	C21—C22—C23—C24	−1.7 (3)
Br^i^—Pd—As—C31	−157.13 (7)	C22—C23—C24—C25	2.0 (3)
C21—As—C11—C16	90.26 (17)	C23—C24—C25—C26	−0.2 (4)
C31—As—C11—C16	−162.82 (16)	C22—C21—C26—C25	2.3 (3)
Pd—As—C11—C16	−32.31 (17)	As—C21—C26—C25	−178.68 (17)
C21—As—C11—C12	−86.42 (19)	C24—C25—C26—C21	−2.0 (4)
C31—As—C11—C12	20.5 (2)	C11—As—C31—C32	−130.34 (18)
Pd—As—C11—C12	151.01 (17)	C21—As—C31—C32	−23.4 (2)
C16—C11—C12—C13	0.3 (3)	Pd—As—C31—C32	105.78 (17)
As—C11—C12—C13	176.92 (19)	C11—As—C31—C36	51.1 (2)
C11—C12—C13—C14	−0.8 (4)	C21—As—C31—C36	158.07 (18)
C12—C13—C14—C15	0.5 (4)	Pd—As—C31—C36	−72.76 (19)
C13—C14—C15—C16	0.1 (4)	C36—C31—C32—C33	−0.9 (3)
C12—C11—C16—C15	0.4 (3)	As—C31—C32—C33	−179.42 (18)
As—C11—C16—C15	−176.38 (18)	C31—C32—C33—C34	−0.3 (4)
C14—C15—C16—C11	−0.6 (4)	C32—C33—C34—C35	1.0 (4)
C11—As—C21—C22	5.24 (18)	C33—C34—C35—C36	−0.6 (4)
C31—As—C21—C22	−102.53 (17)	C34—C35—C36—C31	−0.5 (4)
Pd—As—C21—C22	124.67 (15)	C32—C31—C36—C35	1.3 (4)
C11—As—C21—C26	−173.72 (16)	As—C31—C36—C35	179.81 (19)

Symmetry codes: (i) −*x*+1, −*y*+1, −*z*+1.

### Hydrogen-bond geometry (Å, °)

**Table:**

*D*—H···*A*	*D*—H	H···*A*	*D*···*A*	*D*—H···*A*
C13—H13···Br^ii^	0.95	2.90	3.807 (3)	160
C25—H25···Br^iii^	0.95	2.98	3.914 (3)	168

Symmetry codes: (ii) −*x*+3/2, *y*−1/2, −*z*+3/2; (iii) *x*−1, *y*, *z*.
